# Multimodal Contrast Agent Enabling pH Sensing Based on Organically Functionalized Gold Nanoshells with Mn-Zn Ferrite Cores

**DOI:** 10.3390/nano12030428

**Published:** 2022-01-27

**Authors:** Duong Thuy Bui, Radim Havelek, Karel Královec, Lenka Kubíčková, Jarmila Kuličková, Petr Matouš, Vít Herynek, Jaroslav Kupčík, Darina Muthná, Pavel Řezanka, Ondřej Kaman

**Affiliations:** 1 Institute of Physics, Czech Academy of Sciences, Cukrovarnická 10, 162 00 Prague, Czech Republic; bui@fzu.cz (D.T.B.); kubickol@fzu.cz (L.K.); kulickova@fzu.cz (J.K.); kupcik@fzu.cz (J.K.); 2 Department of Analytical Chemistry, University of Chemistry and Technology, Technická 5, 166 28 Prague, Czech Republic; Pavel.Rezanka@vscht.cz; 3 Department of Medical Biochemistry, Faculty of Medicine in Hradec Králové, Charles University, Šimkova 870, 500 03 Hradec Králové, Czech Republic; havelekr@lfhk.cuni.cz (R.H.); karel.kralovec@upce.cz (K.K.); MuthnaD@lfhk.cuni.cz (D.M.); 4 Department of Biological and Biochemical Sciences, Faculty of Chemical Technology, University of Pardubice, Studentská 573, 532 10 Pardubice, Czech Republic; 5 Faculty of Mathematics and Physics, Charles University, V Holešovičkách 2, 180 00 Prague, Czech Republic; 6 Center for Advanced Preclinical Imaging (CAPI), First Faculty of Medicine, Charles University, Salmovská 2, 120 00 Prague, Czech Republic; petr.matous@lf1.cuni.cz (P.M.); vhery@lf1.cuni.cz (V.H.)

**Keywords:** gold nanoshells, magnetic nanoparticles, surface-enhanced Raman spectroscopy, photoacoustic imaging, transverse relaxivity, cell viability

## Abstract

Highly complex nanoparticles combining multimodal imaging with the sensing of physical properties in biological systems can considerably enhance biomedical research, but reports demonstrating the performance of a single nanosized probe in several imaging modalities and its sensing potential at the same time are rather scarce. Gold nanoshells with magnetic cores and complex organic functionalization may offer an efficient multimodal platform for magnetic resonance imaging (MRI), photoacoustic imaging (PAI), and fluorescence techniques combined with pH sensing by means of surface-enhanced Raman spectroscopy (SERS). In the present study, the synthesis of gold nanoshells with Mn-Zn ferrite cores is described, and their structure, composition, and fundamental properties are analyzed by powder X-ray diffraction, X-ray fluorescence spectroscopy, transmission electron microscopy, magnetic measurements, and UV-Vis spectroscopy. The gold surface is functionalized with four different model molecules, namely thioglycerol, *meso*-2,3-dimercaptosuccinate, 11-mercaptoundecanoate, and (11-mercaptoundecyl)-*N*,*N*,*N*-trimethylammonium bromide, to analyze the effect of varying charge and surface chemistry on cells in vitro. After characterization by dynamic and electrophoretic light scattering measurements, it is found that the particles do not exhibit significant cytotoxic effects, irrespective of the surface functionalization. Finally, the gold nanoshells are functionalized with a combination of 4-mercaptobenzoic acid and 7-mercapto-4-methylcoumarin, which introduces a SERS active pH sensor and a covalently attached fluorescent tag at the same time. ^1^H NMR relaxometry, fluorescence spectroscopy, and PAI demonstrate the multimodal potential of the suggested probe, including extraordinarily high transverse relaxivity, while the SERS study evidences a pH-dependent spectral response.

## 1. Introduction

Magnetic nanoparticles have attracted considerable attention as so-called negative contrast agents for magnetic resonance imaging (MRI), which were popularized mainly through the commercial agents Feridex^®^/Endorem^®^ and Resovist^®^/Cliavist^®^. Specifically, magnetic nanoparticles in the superparamagnetic state have enabled the establishment of an entirely new imaging method–magnetic particle imaging (MPI), which was experimentally demonstrated for the first time in 2005 [1]. The logical effort to combine magnetic imaging techniques, above all MRI, with other imaging modalities in order to increase the accuracy of diagnostics has led to multimodal contrast agents [2].

A plethora of studies, even those accompanied by convincing in vivo experiments, have already presented dual contrast agents for MRI and optical imaging using fluorescence techniques. Moreover, reports combining not just two but several imaging modalities while employing a single contrast agent have not been rare recently, e.g., trimodal imaging by means of MRI, positron emission tomography(PET)/single-photon emission computed tomography (SPECT), and fluorescence techniques [3], or the combination of MRI, X-ray computed tomography (CT), and fluorescence techniques [4]. Much more rare are the reports on trimodal and multimodal contrast agents that would offer reasonable contrast effects both in traditional methods, such as MRI, and in the newly emerging modalities of MPI and photoacoustic imaging (PAI) (see, e.g., [5,6,7,8]).

The last-mentioned method of PAI resolves the traditional optical imaging trade-off between penetration depth and spatial resolution by using the detection of broadband ultrasonic waves. The studied object is irradiated by a short-pulsed laser preferably in the NIR window (620–950 nm), and the locally absorbed light leads to a thermoelastic expansion, which generates ultrasonic waves, here referred to as photoacoustic waves [9]. The initial pressure rise is related to the amount of optical energy absorbed, which also determines the contrast effect in PAI. This technique thus provides the distribution of absorbed optical energy density, and absorption by certain exogenous contrast agents such as gold nanostructures [10] is extremely efficient, enabling the specific visualization of labeled or targeted structures.

Going beyond the limits of state-of-the-art multimodal contrast agents (even functionalized for the molecular recognition of specific structures) means that their diagnostic and analytical potential should be enhanced further. We believe that combining these agents with functionalities that enable the sensing of local physical or chemical properties within the vicinity of the target tissue may enable considerable advances in biomedical research and possibly even in medical diagnostics.

The non-invasive sensing of pH within biological tissues as an added functionality may offer an attractive opportunity for a case study on multimodal contrast agents with applicability extended to the sensing of local physiological conditions at a high spatial resolution. Excellent results on pH sensing in biological systems, including intracellular pH sensing, have been achieved by surface-enhanced Raman spectroscopy (SERS) with gold or silver nanostructures functionalized with pH-dependent SERS reporter molecules, such as *p*-mercaptobenzoic acid (MBA) [11,12,13]. The present study aims to employ virtually the same approach for sensing pH, namely the functionalization of a nanosized SERS substrate with MBA, whose different levels of protonation will be determined by SERS measurements. However, in the present case, the MBA molecules will not be attached to purely plasmonic nanostructures but to a multimodal contrast agent specifically designed for MRI (and possibly MPI), PAI, and fluorescence imaging techniques. The proposed agent will be based on gold nanoshells with silica-coated magnetic cores and organic functionalization with fluorescent and pH-dependent SERS reporter molecules. 

The magnetic cores will be rationally designed to achieve a very high transverse relaxivity, r_2_, which determines the contrast effect in T_2_-weighted MRI, i.e., the performance of a negative contrast agent, all the while requiring superparamagnetic behavior at a physiological temperature. This will be accomplished by using a magnetic phase with high magnetization and a reasonable magnetocrystalline anisotropy in the form of well-defined clusters of ≈ 10 nm crystallites. Such crystallites will acheive Néel relaxation fast enough to still occur in the superparamagnetic regime, although the larger size of the whole cluster will provide much stronger effects in MRI than the well-dispersed 10 nm crystallites [14,15,16]. A suitable candidate is Mn_1-x_Zn_x_Fe_2_O_4_ ferrite with x ≈ 0.4, due to its magnetic properties [17]. In brief, the spinel structure of Mn-Zn ferrite possesses two different metal sublattices, the octahedral one and the tetrahedral one, forming a ferrimagnetic arrangement. Its magnetization is increased by a preferential occupation of the tetrahedral sublattice by diamagnetic Zn^2+^ cations, the substitution with which also facilitates the transition to the superparamagnetic state.

The Mn-Zn ferrite cores will be coated by silica, which provides a suitable dielectric substrate for the attachment of a gold nanoshell and, at the same time, an insulating barrier that encapsulates the magnetic core with a biologically inert and chemically stable material. Then, the gold nanoshells will be synthesized around the silica-coated magnetic cores by applying a rather complex but reliable seed-and-growth method [18]. The final step towards the envisaged multimodal agent will involve the simultaneous covalent functionalization of the gold surface with MBA and 7-mercapto-4-methylcoumarin (MMC) as the fluorescent tag.

In addition to rigorous characterizations of the described contrast agent and its intermediates, the present study will demonstrate those properties which are crucial for MRI, PAI, and fluorescence techniques. A detailed evaluation will also be performed to assess possible cytotoxic and antiproliferative effects in vitro of the crucial component of the suggested platform, i.e., gold nanoshells with silica-coated Mn-Zn ferrite cores. For this purpose, the gold nanoshells will be covalently functionalized with four different model compounds with a mercapto group: (1) thioglycerol, i.e., a small electroneutral molecule providing a hydrophilic surface, (2) *meso*-2,3-dimercaptosuccinate, another small and hydrophilic molecule which predominantly occurs as a monoanion under neutral pH, (3) 11-mercaptoundecanoate, i.e., a surfactant whose aliphatic chain is attached to the gold surface while its anionic head is exposed to water, and (4) (11-mercaptoundecyl)-*N*,*N*,*N*-trimethylammonium bromide, which provides a cationic analogy to the previous compound.

## 2. Materials and Methods

### 2.1. Preparation of Samples

#### 2.1.1. Chemicals

Besides inorganic starting materials and common reagents, the following chemicals were used for the synthesis of gold nanoshells: poly(sodium 4-styrenesulfonate) (PSS) (M_r_ ≈ 70,000, 30 wt% in H_2_O), poly(diallyldimethylammonium chloride) (PDADMAC) (M_r_ ≈ 100,000, 35 wt% in H_2_O), and tetrakis(hydroxymethyl)phosphonium chloride (THPC) (80 wt% in water). Specific organic compounds were also employed for functionalization of gold nanoshells: thioglycerol (TG) (≥97%), meso-2,3-dimercaptosuccinic acid (DMSA) (98%), 11-mercaptoundecanoic acid (MUA) (95%), (11-mercaptoundecyl)-*N*,*N*,*N*-trimethylammonium bromide (MTAB) (≥90%), 4-mercaptobenzoic acid (MBA) (99%), and 7-mercapto-4-methylcoumarin (MMC) (≥97%). All the aforementioned chemicals were purchased from Sigma-Aldrich.

#### 2.1.2. Synthesis of Mn-Zn Ferrite Nanoparticles and Their Encapsulation into Silica 

Mn-Zn ferrite nanoparticles of the composition Mn_0.61_Zn_0.42_Fe_1.97_O_4_ (MZF) and with an expected crystallite size of around 10 nm were prepared under hydrothermal conditions by strictly following the previously published procedure [16]. 

The MZF particles were colloidally stabilized with ammonium citrate and subsequently coated with silica using the Stöber process according to the procedure described in [19] with several minor modifications. In the present study, a different ratio of starting materials was applied, namely 150 mg of MZF particles and 2.94 mL of tetraethoxysilane, and the encapsulation into silica was carried out in a mixture of 300 mL of 96 vol% ethanol, 40 mL of water, and 20 mL of 29 wt% ammonium hydroxide. After thorough purification of the raw product (repeated redispersion in ethanol and later in water followed by centrifugation at 7000 rcf for 40 min), the silica-coated particles were subjected to differential centrifugation in water at 1864 rcf for 15 min, and the supernatant containing the fine fraction was collected as the final product while the residue was discarded. The so-obtained aqueous suspension of silica-coated Mn-Zn ferrite nanoparticles (MZF@sil) was adjusted to a final volume of 90 mL. The ferrite concentration was determined by magnetometric analysis to be 2.94 mmol(f.u.) L^−1^, where f.u. stands for the formula unit of Mn_0.61_Zn_0.42_Fe_1.97_O_4_, which corresponds to a yield, primarily of the fractionation, of 41% with respect to starting MZF particles.

#### 2.1.3. Preparation of Gold Nanoshells with Silica-Coated Ferrite Cores

The gold nanoshells enclosing silica-coated ferrite cores (MZF@sil@Au) were prepared using a multistep procedure that consisted of (i) the formation of a PDADMAC/PSS polyelectrolyte multilayer on the silica surface of MZF@sil via a layer-by-layer self-assembly technique [20], (ii) adsorption of ultrafine gold seeds synthesized according to Duff [21], (iii) the growth of the gold seeds to coalescence by reduction of a suitable soluble gold(III) precursor, and (iv) size fractionation. The whole procedure was based on previously published reports [18,22] but involved significant modifications, and thus the full details are provided below.

At first, ultrafine gold nanoparticles were prepared using the Duff method, i.e., under alkaline conditions by employing THPC, in a partially hydrolyzed form, as the reducing agent of aqueous HAuCl_4_ and primary nucleating agent of the resulting gold [21]. Specifically, 2.4 mL of 25.4 mM HAuCl_4_ was added into a round-bottom flask containing 55 mL of water, 1.8 mL of 0.2 M NaOH, and 1.2 mL of 67 μM THPC, while magnetic stirring was applied. The brownish mixture was further stirred for 2 h, and the resulting gold colloid was purified as follows. The mixture was concentrated in vacuo to ≈10 mL, and its colloidal stability was impaired by the addition of ≈35 mL of acetone. The nanoparticles were separated by exhaustive centrifugation (7000 rcf for 1 h) and were redispersed in pure water. Residual acetone was removed in vacuo, and the suspension was adjusted to a final volume of 60 mL.

Simultaneously, the polyelectrolyte multilayer was deposited on the MZF@sil nanoparticles to alter the zeta potential of their surface from negative to positive values. In the first step, 4 mL of the MZF@sil suspension with a ferrite concentration of 1.34 mmol(f.u.) L^−^^1^, 2 mL of 0.75 M NaCl, and 5 μL of PDADMAC (35 wt%) were mixed in a round-bottom flask and agitated for 15 min in an ultrasonic bath tempered to room temperature. The particles were then separated by exhaustive centrifugation (15,800 rcf for 5 min), washed with water in three cycles (centrifugation at 15,800 rcf for 5 min in each cycle) and redispersed in 4 mL of water. In the subsequent steps, the procedure was repeated but the PSS (30 wt%) was used instead of the PDADMAC polyelectrolyte in every even step until a sequence of five layers with the composition (PDADMAC-PSS)_2_-PDADMAC was achieved. The final purification also involved three washing cycles in water (centrifugation at 15,800 rcf for 5 min in each cycle), and the particles were redispersed in 4 mL of water.

The so-obtained particles with the polyelectrolyte multilayer were mixed with 60 mL of the freshly prepared (on the same day) ultrafine gold nanoparticles, and the mixture was stirred overnight in an ultrasonic bath tempered to room temperature. The mixture was then subjected to differential centrifugation at 4193 rcf for 30 min, which led to the sedimentation of MZF@sil particles decorated with gold nanoparticles while the excess free gold colloid remained in the supernatant. The sediment was subjected to six further cycles of the same differential centrifugation in pure water, whereas the corresponding supernatants were discarded. The decorated product was adjusted to the final volume of 40 mL.

One day prior to the growth of the gold nanoshells, the so-called K-gold solution was prepared by mixing 7.5 mL of 25.4 mM HAuCl_4_ with 135 mg of K_2_CO_3_ dissolved in 42.5 mL of water and by subsequent aging of the solution in a fridge overnight. For the synthesis of gold nanoshells, 37.5 mL of the decorated particles were mixed with 562 mL of water and 150 mL of 3 mM L-ascorbic acid in a two-neck round-bottom flask placed in an ultrasound bath tempered to room temperature. A Teflon mechanical stirrer was introduced, and the mixture was subjected to both mechanical and ultrasound agitation. The gradual growth of the gold seeds to coalescence was achieved by the continuous addition of 50 mL of the K-gold for 150 min via a syringe pump. Thereafter, the raw product was separated by exhaustive centrifugation (7000 rcf for 15 min) and washed twice with water (centrifugation at 7000 rcf for 15 min in each cycle). Finally, differential centrifugation at 116 rcf for 5 min was applied, whereby the fine fraction of gold nanoshells was isolated in the supernatant while the heavy fraction was discarded with the residue.

#### 2.1.4. Organic Functionalization of Gold Nanoshells

All organic functionalizations were generally carried out by transferring the gold nanoshells into a suitable solvent, where they were allowed to bind respective mercaptans, and the resulting products were subjected to purification by several washing cycles. In the case of functionalization with DMSA and MUA, a neutralization step with a dilute NaOH was involved to convert the carboxylic acids to sodium carboxylates.

Specifically, the MZF@sil@Au particles were collected from the aqueous suspension by exhaustive centrifugation (7000 rcf for 10–15 min, the same centrifugation conditions applied in all subsequent separation steps) and were washed using either acetonitrile or ethanol. The particles were then redispersed in a 1 mM ethanolic solution of one of the following compounds: TG, DMSA, MUA, or MTAB, or in an acetonitrile solution of MBA and MMC in a molar ratio of 5:1 and a total concentration of 1–50 mmol L^−1^ (the higher concentration was employed while preparing the sample for the SERS study and temperature-variable ^1^H relaxometry). The suspensions were mixed in an ultrasonic bath for 5–14 h, and then the particles were separated by exhaustive centrifugation. Thorough purification by ethanol/acetonitrile and water followed. In the case of the DMSA and MUA-functionalized gold nanoshells, after washing with ethanol and water, the product was further washed twice with 0.01 M NaOH and an additional three washing cycles in water followed. The functionalized gold nanoshells are denoted as MZF@sil@Au-x where x is the abbreviation of the model compound.

### 2.2. Physical and Chemical Characterizations

The phase composition, crystal structure, and the mean size of crystallites, d_XRD_, of the bare ferrite nanoparticles and final gold nanoshells were analyzed by powder X-ray diffraction (XRD) with Cu Kα radiation. The measurements were carried out at room temperature on a Bruker D8 Advance diffractometer, and the patterns were analyzed using the Rietveld method in the FullProf program. The Thompson-Cox-Hastings pseudo-Voigt function was employed to resolve strain and size contributions to the line broadening, while the instrumental function was experimentally determined based on a strain-free LaB_6_ standard.

The overall chemical composition of the functionalized gold nanoshells MZF@sil@Au-MBA+MMC was determined using X-ray fluorescence spectroscopy (XRF) by using the Orbis® PC Micro-XRF spectrometer by EDAX, with an Rh X-ray tube (40 kV) and an incident beam with the diameter of 30 µm.

The size and morphology of particles were studied using a transmission electron microscope FEI Tecnai G^2^ 20 equipped with a thermionic LaB_6_ cathode and with an acceleration voltage of 200 kV. The size distribution of particles was determined for MZF@sil nanoparticles, Au seeds on the surface of the decorated intermediate, and for MZF@sil@Au nanoshells based on image analysis of micrographs by using the ImageJ software [23]. For 300 particles in each case, two representative diameters of each particle were collected and their average d_TEM_ was employed in further analyses (histogram, mean diameter d_mean_ and its standard deviation SD).

The colloidal stability and hydrodynamic size of both bare and functionalized gold nanoshells were probed using dynamic light scattering (DLS), which was measured on highly dilute suspensions in pure water tempered to 25 °C by using a Malvern Zetasizer Nano-ZS instrument. In addition, the DLS measurement was carried out for MZF@sil@Au-MBA+MMC nanoshells in 10 vol% fetal bovine serum (FBS, from Sigma-Aldrich) to probe the colloidal behavior under conditions more similar to physiological ones. Out of the three/six measurements of each sample, representative intensity distribution data are presented along with the respective Z-average values, d_hydro,Z_. The same instrumentation was also used to measure the zeta potential of gold nanoshells functionalized with the four model compounds (TG, DMSA, MUA, and MTAB) using the electrophoretic light scattering (ELS) method. For the samples with different model functionalization, the dependence of zeta potential on pH was determined based on a series of aqueous suspensions whose pH was carefully adjusted using diluted NaOH or HCl.

Dilute aqueous suspensions of bare and MZF@sil@Au-MBA+MMC nanoshells were also subjected to UV-Vis absorption spectroscopy in order to analyze the surface plasmon resonance (SPR) of the gold nanoshells and possible effects of the given surface functionalization. The spectra were measured in quartz cuvettes on an Agilent Cary 300 UV-Vis spectrophotometer.

The magnetic behavior of the gold nanoshells, the silica-coated ferrite particles, and also the bare ferrite particles was studied using SQUID magnetometry in DC fields by using a Quantum Design MPMS XL system. Besides measurements of M-H dependences at 5 K and 300 K, the blocking behavior of magnetic nanoparticles in the samples was analyzed using temperature-variable measurements of susceptibility, which were carried out in zero-field-cooled (ZFC) and field-cooled (FC) regimes in the temperature range of 5–390 K by using the probe field of 2 mT. Prior to the cooling in zero field, a controlled quench of the solenoid was applied to remove any remnant fields in the superconducting winding.

The concentration of ferrite nanoparticles in the suspension of silica-coated particles and all different suspensions of functionalized gold nanoshells was determined using magnetometric analysis. An aliquot of 800 μL (MZF@sil) or 200 μL (gold nanoshells) was transferred onto a small piece of Teflon tape spread on a clock glass and was precisely weighed and dried carefully. The dry sample was weighed again and mounted in a gelatine capsule for the magnetic measurements. By considering the magnetization of bare ferrite cores, the ferrite content in the aliquot was calculated based on the magnetic moment of the sample measured at 5 K in the magnetic field of 0.3 T, where the diamagnetic contribution was insignificant (the contribution of the mounting material was carefully checked/corrected).

### 2.3. Biological Studies

#### 2.3.1. Cell Culture and Culture Conditions

The biological experiments employed human breast carcinoma cell line MCF-7, which was purchased from the European Collection of Cell Cultures (ECACC) and cultured in accordance with the provider’s culture method guidelines. The stock cells were grown in 75 cm^2^ tissue culture flasks (TPP) and maintained under standard cell culture conditions at 37 °C in a humidified incubator in an atmosphere of 5% CO_2_ and 95% air. The cells were passaged every 2 to 3 days to obtain exponential growth, and only cells within the maximum range of 20 passages were used for this study.

#### 2.3.2. Real-Time Cytotoxicity Assay

The cytotoxicity of the MZF@sil@Au-DMSA, MZF@sil@Au-TG, MZF@sil@Au-MUA, and MZF@sil@Au-MTAB was assessed against MCF-7 cells using the xCELLigence RTCA SP (real-time cell analysis, single-plate) system (Roche Diagnostic), allowing the label-free dynamic monitoring of cell events in real-time. The principle of the system is to monitor the changes in electrode impedance induced by the interaction between tested cells and electrodes [24,25]. The xCELLigence system was connected and tested using resistor plate verification before the RTCA SP station was placed inside the incubator at 37 °C and 5% CO_2_. Background measurements were taken by adding 100 μL of appropriate medium to the wells of the E-Plate 96. A cell suspension (90 μL) at a cell density of 11,500 cells per well was added to each well of the E-plate 96. The MCF-7 cell proliferation was dynamically monitored at the 15 min interval. Approximately 22 h later, when the cells were in the log growth phase, the cells were exposed in tetraplicates to 10 µL of sterile deionized water with MZF@sil@Au-DMSA, MZF@sil@Au-TG, MZF@sil@Au-MUA, or MZF@sil@Au-MTAB nanoshells to obtain the final desired concentration in each well. Negative controls received sterile deionized water for cell cultures (Lonza), whereas cells treated with 5% DMSO and 0.5 μM doxorubicin were used as positive controls. The incubation of the treated cells lasted 72 h. The cell status and the cytotoxic effect were plotted using the characteristic cell index (CI)-time profile, and the growth curves were normalized to 1 at the time point of the treatment. Evaluations were performed by using the RTCA 1.2.1 software.

#### 2.3.3. Proliferation and Viability Measurement Using Trypan Blue Exclusion Test

Cell proliferation and the viability of MCF-7 cells were monitored for 48 h after the treatment with 1.4, 2.7, and 5.3 µmol(f.u.) L^−1^ of MZF@sil@Au-DMSA, MZF@sil@Au-TG, MZF@sil@Au-MUA, or MZF@sil@Au-MTAB. Cells treated with 1 µM doxorubicin were used as a positive control and cells treated with sterile deionized water as a negative control. Cell membrane integrity was determined using the trypan blue exclusion technique, by mixing 10 μL of 0.4% trypan blue and 10 μL of cell suspension. Cell counts were carried out using a Bürker chamber and a Nikon Eclipse E200 light microscope.

#### 2.3.4. Cell Cycle Distribution and Internucleosomal DNA Fragmentation Analysis

Cells employed in the cell-cycle distribution analysis, i.e., cells incubated with the highest concentration of 5.3 µmol(f.u.) L^−1^ of functionalized nanoshells and both positive and negative controls, were harvested by trypsinization, collected, washed with ice-cold PBS, and fixed with 70% ethanol. The cells were centrifuged to remove ethanol and washed again with ice-cold PBS. In order to detect low-molecular-weight fragments of DNA, the cells were incubated for 5 min at room temperature in a buffer (192 mL of 0.2 M Na_2_HPO_4_ + 8 mL of 0.1 M citric acid, pH 7.8) and then labelled with propidium iodide in Vindelov’s solution for 1 h at 37 °C. The DNA content was determined by using a CytoFLEX LX flow cytometer by Beckman Coulter with an excitation wavelength of 488 nm. The data were analyzed using Kaluza Analysis 2.1 software.

#### 2.3.5. Statistical Analysis

In this study, all values were expressed as arithmetic means with SD calculated from triplicates, unless otherwise noted. In experiments with parametric variables, significant differences between the groups were analyzed using the Student’s *t*-test and a *p*-value ≤ 0.05 was considered significant.

### 2.4. ^1^H Relaxometry, Photoacoustic Imaging, and Fluorescence Properties

To evaluate the suitability of the present gold nanoshells with Mn-Zn ferrite cores as contrast agents for MRI, a ^1^H NMR relaxometry study was carried out on aqueous suspensions of MZF@sil@Au-MBA+MMC particles in the magnetic field of 0.47 T (^1^H Larmor frequency of 20 MHz). The stock suspension was diluted to concentrations of the ferrite formula units of 0.032, 0.0128, and 0.0064 mmol(f.u.) L^−1^, and the resulting suspensions were measured at 23 °C with a Bruker MiniSpec mq20 relaxometer. Longitudinal relaxation times, T_1,_ were probed using a saturation recovery sequence (10 points with varying recovery times in the range of 100 to 10,000 ms). Transverse relaxation times, T_2_, were determined using a Carr-Purcell-Meiboom-Gill (CPMG) multi-spin echo sequence (echo spacing TE = 2 ms, repetition time TR = 10 s, number of acquisitions NA = 4). Relaxivities r_1_ and r_2_ were calculated by using a linear fit of the dependence of relaxation rates R_1_ and R_2_ (reciprocal values of T_1_ and T_2_), respectively, on the molar concentration of the ferrite. In addition, the temperature dependence of r_2_ was determined for MZF@sil@Au-MBA+MMC particles based on measurements of T_2_ on a suspension with a concentration of 0.0163 mmol(f.u.) L^−1^ whose temperature was controlled by an external water bath and monitored directly in the suspension.

PAI experiments were performed on the aqueous suspensions of MZF@sil@Au-MBA+MMC particles at the concentration of 0.64 mmol(f.u.) L^−1^. An aliquot of the suspension was injected into a silicone tube (inner/outer diameter 0.5/2.5 mm) and the tube was submerged in bubble-free water. Scanning was carried out by using a preclinical bimodal imaging platform Fujifilm VisualSonics Vevo 3100/LAZR-X, which combines high-frequency ultrasound and photoacoustic imaging. The photoacoustic spectra were acquired with an MX400 transducer (20–46 MHz, 50 µm axial resolution) equipped with a jacket for inserting optical fiber bundles (14 mm wide). The spectra were measured in the near-infrared range of 680–970 nm with laser pulse energy 36 mJ; the presented spectrum is an average of five scans. The raw data were processed by the dedicated software Vevo LAB by Fujifilm VisualSonics.

The fluorescence properties of the MZF@sil@Au-MBA+MMC particles were probed using spectrofluorimetry at room temperature on an aqueous suspension with a concentration of ≈0.02 mmol(f.u.) L^−1^ whose pH was adjusted to 7. The spectra were recorded in a quartz cuvette with an Agilent Cary Eclipse fluorescence spectrophotometer. The excitation scan was measured while monitoring emission at λ_em_ = 375 nm and the emission scan was recorded with excitation at λ_ex_ = 314 nm.

### 2.5. Surface-Enhanced Raman Spectroscopy Study

A series of aqueous suspensions of MZF@sil@Au-MBA+MMC nanoparticles was prepared with varying pH adjusted in the range of 3–10 via the addition of dilute HCl or NaOH solutions. Their SERS spectra were measured by an i-Raman® Plus spectrometer with an excitation wavelength of 785 nm and laser power of 170 mW. Each spectrum was obtained by the accumulation of 100 scans with an acquisition time of 1 s per scan. For the analysis of the pH-dependent spectral response, only the region of 1235–975 cm^−1^ was processed by applying the automatic baseline correction in the OMNIC software and then the normalization of the spectra to the band at 1056 cm^−1^.

## 3. Results and Discussion

### 3.1. Synthesis and Fundamental Properties of Gold Nanoshells

**Figure 1:**
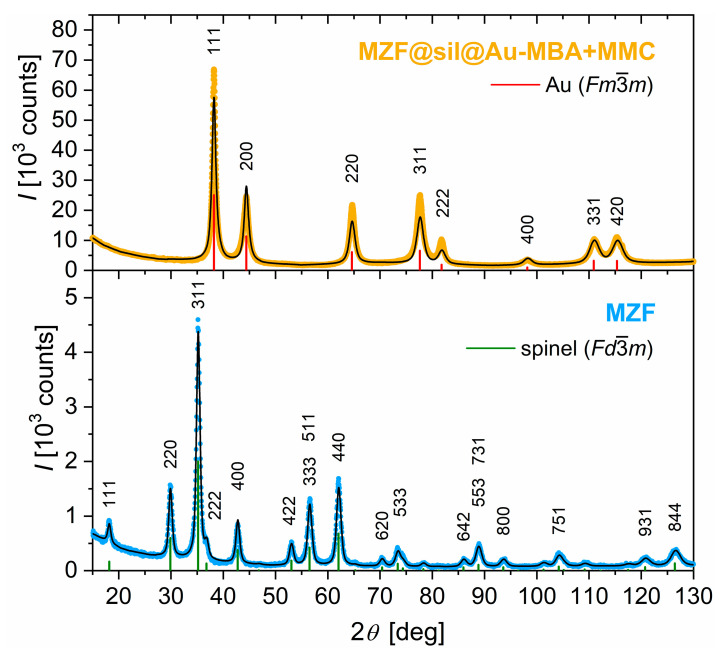
Powder XRD patterns of the starting Mn_0.61_Zn_0.42_Fe_1.97_O_4_ nanoparticles (MZF) and the final organically functionalized gold nanoshells with silica-coated ferrite cores (MZF@sil@Au-MBA+MMC). The measured data are complemented with the calculated patterns (black lines) based on the Rietveld refinement of the spinel ($Fd\bar{3}m$) and gold ($Fm\bar{3}m$ ) structures (original structure data retrieved from the Inorganic Crystal Structure Database under the collection codes 98553 and 64701). The diffraction lines of the refined structures are depicted by green and red verticals, respectively.

The XRD analysis confirmed the single-phase character of hydrothermally prepared Mn_0.61_Zn_0.42_Fe_1.97_O_4_ nanoparticles and their typical cubic spinel structure with $Fd\bar{3}m$ symmetry (see the XRD pattern in the lower panel of Figure 1). The lattice parameter at room temperature was refined to a = 8.4542(2) Å, which is practically identical to a = 8.4576(4) Å reported for the given composition in the original study [16]. The mean size of crystallites was evaluated based on the broadening of the diffraction lines by using the Rietveld method to d_XRD_ = 12 nm, which is also consistent with the value of 11 nm determined for the prototypical product in [16].

Interestingly, the XRD pattern of the gold nanoshells (see the upper panel of Figure 1) is entirely dominated by broadened diffraction lines of the cubic gold with $Fm\bar{3}m$ symmetry, while the diffraction lines of the spinel phase are not discernible, which, however, can be expected taking into account the ratio of gold and ferrite particles (12 mg + 37.5 mg Au via the ultrafine colloid and K-gold, respectively, per 1.2 mg MZF) employed in the synthesis and the magnitudes of scattering factors of Au vs. elements that form the ferrite. The actual content of ferrite in the nanoshells was estimated based on XRF, which provided a weight ratio of Mn-Zn ferrite: Au = 0.017 or 0.013 based on the ratio of Au and Fe or Mn lines, respectively, although significant uncertainties related to the quantification due to the low content of the ferrite have to be considered.

The lattice parameter of gold nanoshells was refined to a = 4.0765(1) Å, which is highly comparable with values reported for gold of high purity in bulk form, e.g, a = 4.07894(5) Å according to [26]. The significant broadening of its diffraction lines evidenced the nanocrystalline character, and the Rietveld analysis with an isotropic model for gold crystallites provided d_XRD_ = 7 nm.

**Figure 2:**
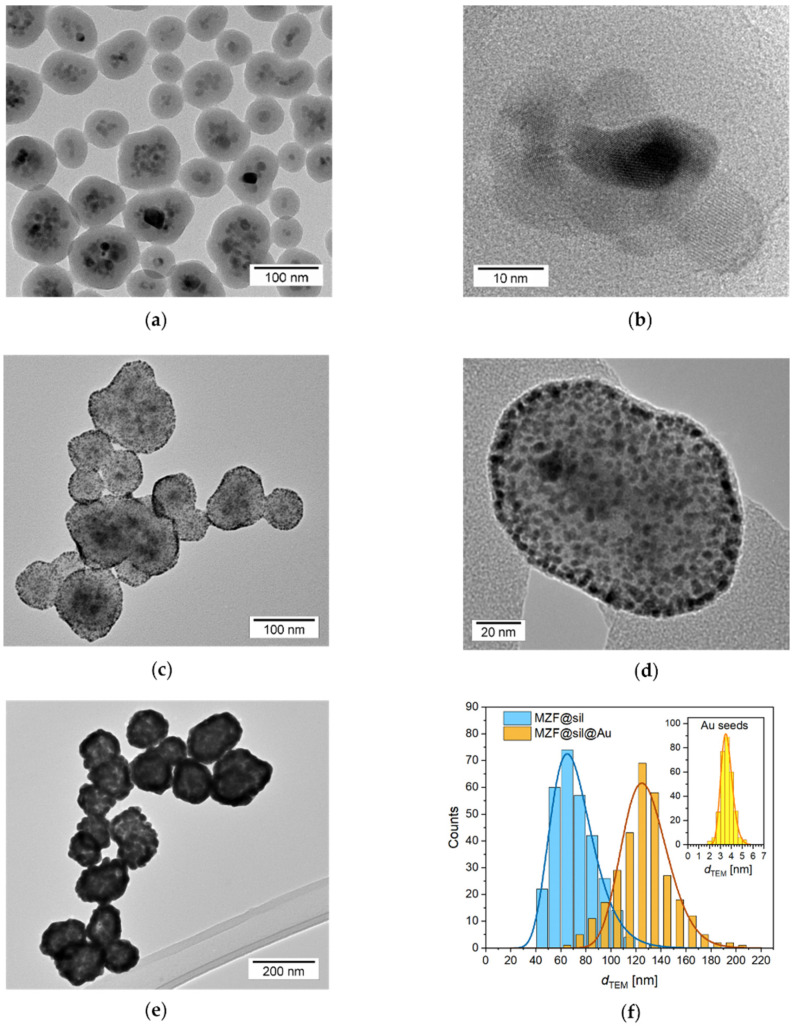
Transmission electron micrographs and size distribution of nanoparticles: (**a**) MZF@sil; (**b**) HRTEM detail of a magnetic core of MZF@sil particles; (**c**,**d**) MZF@sil nanoparticles decorated with gold seeds; (**e**) MZF@sil@Au; (**f**) histograms of MZF@sil and MZF@sil@Au sizes, with the inset showing the histogram of Au seeds on the surface of the decorated intermediate; the histograms were fitted with a log-normal distribution function.

Conclusive data on the actual size of particles and their morphology were provided by the TEM study accompanied by a thorough analysis of size distribution. Representative transmission electron micrographs of the silica-coated ferrite nanoparticles, the subsequent intermediate decorated with fine gold seeds, and the final gold nanoshells are shown in Figure 2a–e, while Figure 2f presents histograms of sizes for silica-coated particles, final gold nanoshells, and also gold seeds on the silica surface of the intermediate. 

A striking feature of the MZF@sil particles is the structure of their magnetic cores that were predominantly formed by small clusters of ferrite nanocrystallites and were encapsulated as a whole by a continuous silica shell. The crystallinity of individual ferrite nanoparticles and their rather random orientation in the observed clusters were evidenced by the lattice fringes visible in the HRTEM mode (Figure 2b). In contrast, the silica shell was amorphous, and it exhibited a smooth surface and uniform thickness of 20 nm (with a standard deviation of just 2.0 nm). The mean diameter of silica-coated particles was d_mean_(MZF@sil) = 72 nm, and the examined set was described by a standard deviation of 17 nm. Considering the mentioned shell thickness, the mean size of the whole magnetic cores formed by the clusters of ferrite crystallites was ≈32 nm.

The gold-decorated intermediate was characterized by highly dense and homogeneous coverage of the silica surface with fine gold seeds, which was enabled by a successful formation of a thick polyelectrolyte multilayer terminated by the cationic PDADMAC. This multilayer altered the negatively charged surface of silica to a positively charged one, thus allowing electrostatic adsorption of the negatively charged gold seeds [27]. The mean size of the attached gold nanoparticles was determined to be d_mean_(Au) = 3.6 nm with a standard deviation of 0.5 nm, which is in agreement with gold nanoparticles reported by Duff [21].

The final gold nanoshells showed typical morphology with a rather rough surface and a somewhat porous structure clearly reminiscent of the seed-and-growth procedure. The gold nanoshells exhibited the mean diameter of d_mean_(MZF@sil@Au) = 127 nm, and the analyzed set was described by a standard deviation of 22 nm. The comparison of this value with d_mean_(MZF@sil) suggests that the mean thickness of gold nanoshells was ≈27 nm in a rough spherical approximation.

**Figure 5:**
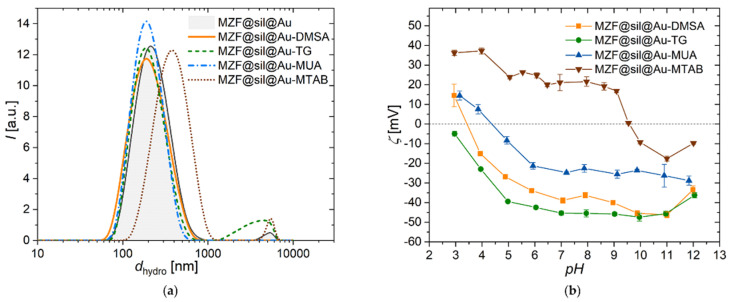
Dynamic and electrophoretic light scattering studies on gold nanoshells functionalized with four different model molecules (DMSA, TG, MUA, MTAB): (**a**) The intensity-weighted hydrodynamic size distribution of gold nanoshells functionalized with the four model compounds, measured on dilute aqueous suspensions by DLS; (**b**) dependence of zeta potential on pH measured by ELS; the error bars show 95% confidence intervals based on repeated measurements.

The DLS measurement of bare gold nanoshells in a dilute aqueous suspension revealed a Z-average hydrodynamic size of d_hydro,Z_(MZF@sil@Au) = 208 nm and polydispersity index of pdi = 0.201. The intensity distribution of the hydrodynamic size of bare nanoshells is included in Figure 5a. The difference between the DLS and TEM sizes cannot be attributed just to a hydration layer around gold nanoshells, although the hydration shell definitely increases the hydrodynamic size, and certain aggregation has to be taken into account. Importantly, the DLS of MZF@sil@Au nanoshells did not reveal colloidal instability, and the results were highly repeatable.

**Figure 3:**
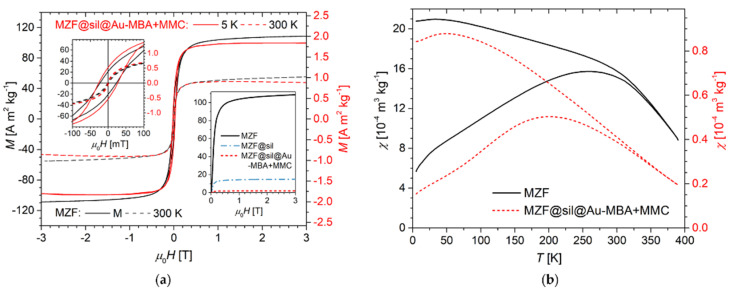
Magnetic properties: (**a**) Hysteresis loops of bare ferrite nanoparticles (left axis) and final gold nanoshells (right axis) at temperatures of 5 K and 300 K (the scale of the right axis is adjusted so that the low-temperature curves of both samples coincide at μ_0_H = 0.3 T). The low-field details of magnetization curves are depicted in the upper left inset. The lower right inset shows the virgin curves for bare ferrite nanoparticles, silica-coated intermediate, and final gold nanoshells at 5 K. (**b**) ZFC-FC mass susceptibilities, χ_ZFC_ and χ_FC_, of bare ferrite nanoparticles (left axis) and gold nanoshells (right axis) in the magnetic field of 2 mT.

Hysteresis loops measured at low and room temperatures on the bare MZF particles and final gold nanoshells are presented in Figure 3a together with the respective low-temperature virgin curves, among which also the silica-coated particles are included. The bare MZF particles exhibited high magnetization, of 102.6 A m^2^ kg^−1^ at 5 K and 55.3 A m^2^ kg^−1^ at 300 K, in the magnetic field of 3 T, which is in excellent agreement with the magnetization data reported in a thorough study on the structure and magnetic properties of hydrothermally prepared Mn-Zn ferrite nanoparticles [17]. The MZF bare particles were characterized by a coercivity of μ_0_H = 25 mT at 5 K and a practically anhysteretic curve at 300 K, where the MZF particles were either in the fully superparamagnetic regime on the timescale of DC magnetometry or exhibited a coercivity lower than the experimental limit of the measurement (given by remnant fields in the SQUID superconducting solenoid).

The gold nanoshells showed a similar response to the external magnetic field given by their ferrite cores, but two distinct features should be commented on. First, the magnitude of magnetization per mass of the material was considerably decreased since the magnetic cores were coated by robust silica and gold layers, both diamagnetic, that strongly diluted the ferrimagnetic phase. Second, the high-field region of the M-H dependence measured on the nanoshells exhibited a different slope compared to a clear linear paraprocess in the bare MZF particles. Actually, the slope of the high-field magnetization curve in the former samples was decreased by the small but already significant diamagnetic susceptibility of the coating materials.

By comparing the magnetization values of the bare MZF particles and the coated samples in a magnetic field above the hysteresis but below a significant contribution of the diamagnetic component, the weight content of the ferrite phase in the products can be roughly estimated. Specifically, at 5 K in the magnetic field of μ_0_H = 0.3 T, the MZF, MZF@sil, and MZF@sil@Au-MBA+MMC samples were characterized by a magnetization of 91.8, 13.1, and ≈2.1 A m^2^ kg^−1^, respectively (see the inset in Figure 3a), which leads to the content of Mn-Zn ferrite of 14 wt% in the MZF@sil and of only ~2 wt% in the MZF@sil@Au-MBA+MMC sample, which provides an unambiguous explanation for the missing diffraction lines of the spinel structure in the XRD pattern. Since the weight fraction of the organic component (a monolayer of low-molecular MBA and MMC on the gold surface) in the MZF@sil@Au-MBA+MMC particles was negligible, their overall composition can be roughly described as: ~2 wt% of Mn_0.61_Zn_0.42_Fe_1.97_O_4_, ~14 wt% silica, and ~84 wt% Au.

Regarding the thermal stability of the ferrimagnetic ordering in Mn-Zn ferrite nanoparticles of the given composition and size, the transition to the paramagnetic state occurs at temperatures much higher than room temperature, even above the experimental limit of the set-up used in the present study, and was estimated to be ≈425 K in the previous report [28]. Therefore, the ZFC-FC study in Figure 3b provides primarily an insight into the blocking behavior of ferrite nanocrystallites. The irreversibility temperature, i.e., the bifurcation of the ZFC-FC susceptibility curves, was at ≈330–350 K, indicating the temperature where the superparamagnetic state was achieved within the whole sample. However, the predominant fraction of ferrite nanoparticles was already in the superparamagnetic state at room temperature. The comparison of the ZFC maxima between bare ferrite particles and the gold nanoshells reveals a shift in the distribution of blocking temperatures of ferrite nanoparticles to lower temperatures upon their encapsulation, which can be rationalized based on the suppression of dipolar interparticle interactions by the diamagnetic barriers in the coated sample [29].

**Figure 4:**
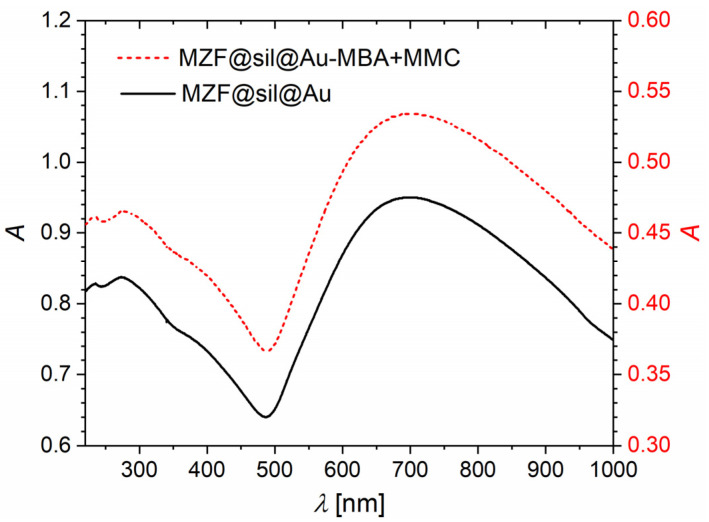
UV-Vis spectra of bare gold nanoshells (MZF@sil@Au, left axis) and gold nanoshells functionalized with the combination of MBA and MMC (MZF@sil@Au-MBA+MMC, right axis).

Figure 4 shows UV-Vis absorption spectra of bare gold nanoshells and their counterparts functionalized with a combination of 4-mercaptobenzoic acid and 7-mercapto-4-methylcoumarin. Both the spectra are dominated by a broad absorption band with a maximum at ≈700 nm, which is given by the SPR of gold nanoshells. The considerable width of the SPR band originated in the broad size distribution of nanoshells and was probably further extended by the aggregation. Nevertheless, the significant extension of the band to NIR offers the advantage of a higher penetration depth in biological tissues and promising optical properties for PAI. The UV-Vis spectrum was not significantly affected upon the applied functionalization. Actually, the absorption due to the SPR in gold nanostructures is by orders of magnitude stronger than the absorption of organic chromophores such as MBA or MMC, and thus no additional absorption features emerged in the spectrum of the functionalized product. Importantly, it follows that the functionalization did not interfere with the SPR band, and the applied procedure neither impaired the gold nanostructure nor led to spectrally significant aggregation.

### 3.2. Gold Nanoshells Functionalized with Model Organic Molecules

The DLS measurements of the hydrodynamic size of gold nanoshells functionalized with the four model molecules, supplemented by the measurement of gold nanoshells prior to functionalization, are depicted in Figure 5a. The distribution data and Z-average values suggest that the samples modified with DMSA, TG, and MUA were roughly comparable with the initial nanoshells (MZF@sil@DMSA, MZF@sil@Au-TG, and MZF@sil@Au-MUA were characterized by d_hydro,Z_ = 184, 208, and 182 nm, and pdi = 0.172, 0.249, and 0.161, respectively), whereas the functionalization with the cationic surfactant MTAB led to a considerable increase in the hydrodynamic size (d_hydro,Z_ ≈ 396 nm and pdi = 0.298), presumably due to aggregation enhanced by the given functionalization.

Figure 5b shows the dependence of the zeta potential on pH for all samples functionalized with the model molecules. The MZF@sil@Au-MTAB nanoshells showed a zeta potential of 20 mV at neutral pH and an isoelectric point pI = 9.6, which confirms the corresponding functionalization. Its anionic counterpart MZF@sil@Au-MUA with long aliphatic carboxylate showed practically inverse behavior with a zeta potential of −23 mV at neutral pH and an isoelectric point pI = 4.4. The samples MZF@sil@Au-TG and MZF@sil@Au-DMSA exhibited a strongly negative zeta potential of −45 and −39 mV at neutral pH, which explains their colloidal stability due to strong coulombic repulsion, and pI ≈ 2.7 and pI = 3.4, respectively. The pH dependence of the zeta potential of the TG-functionalized nanoshells was governed by the native behavior of MZF@sil@Au particles since the thioglycerol moiety in water does not participate significantly in any ionization equilibria, at least not at moderate pH. This explanation is also supported by the measurement of the pH dependence of the zeta potential of native gold nanoshells with silica-coated magnetic cores in [22].

**Figure 6:**
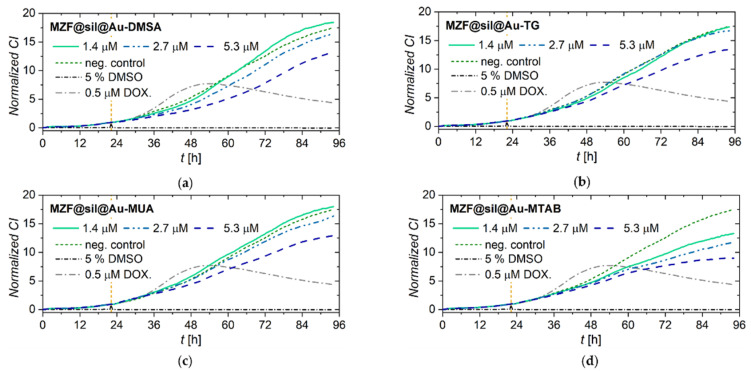
Growth kinetics of human MZF-7 breast carcinoma cells incubated with functionalized gold nanoshells as monitored by the xCELLigence system: Cells treated with (**a**) MZF@sil@Au-DMSA; (**b**) MZF@sil@Au-TG; (**c**) MZF@sil@Au-MUA; (**d**) MZF@sil@Au-MTAB of given concentrations. The negative (vehicle) control was treated with sterile deionized water, whereas the cells treated with 5% DMSO and 0.5 μM doxorubicin were used as positive controls. The yellow vertical line marks the time point of the treatment.

The viability and proliferation of MCF-7 cells incubated with gold nanoshells functionalized with four different model compounds (DMSA, TG, MUA, MTAB) were assessed in real-time by using the label-free xCELLigence system (Figure 6). The xCELLigence system allows the continuous monitoring of cell adhesion, viability, and proliferation based on measurement of impedance, which is displayed as normalized cell index (CI) values. MCF-7 cells treated with MZF@sil@Au-DMSA, MZF@sil@Au-TG, and MZF@sil@Au-MUA nanoshells at lower concentrations of the ferrite 1.4 and 2.7 µmol(f.u.) L^−1^ (i.e., weight concentration of the whole nanoshells ~14 and ~28 µg mL^−1^ according to magnetometry) proliferated in parallel with cells in the negative control, as indicated by the increase in CI values. Only a minor decrease in the CI compared to the negative control was observed for cells treated with MZF@sil@Au-MTAB nanoparticles. At the highest concentration of the nanoshells, 5.3 µmol(f.u.) L^−1^ (≈55 µg mL^−1^ of the whole nanoshells), the proliferation of the cells was slightly impeded, which can be ascribed to the much higher amount of the material used for the incubation, as the ferrite forms only ≈2 wt% of the gold nanoshells (see above).

**Figure 7:**
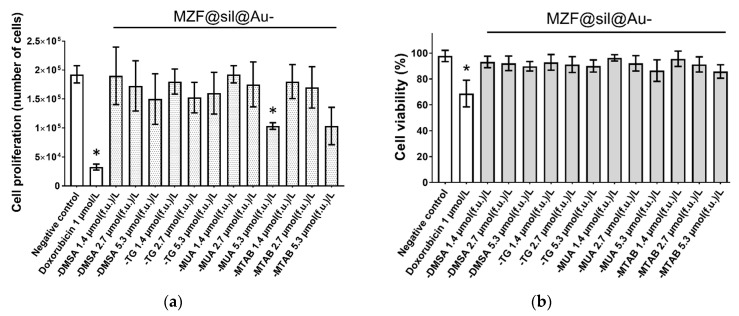
The effect of MZF@sil@Au-DMSA, MZF@sil@Au-TG, MZF@sil@Au-MUA, or MZF@sil@Au-MTAB on the (**a**) proliferation and (**b**) viability of MCF-7 cells. Changes in the proliferation and viability were monitored using the trypan blue exclusion test 48 h after the treatment. Results are shown as mean ± SD from three experiments; the asterisk marks results significantly different from the control (*p* ≤ 0.05). Cells treated with 1 µM doxorubicin and sterile deionized water were used as a positive and negative control, respectively.

To further explore the effect of the functionalized gold nanoshells on cell proliferation rate and viability, trypan blue dye exclusion staining was performed after the MCF-7 cells had been treated for 48 h with the nanoshells at concentrations in the range of 1.4–5.3 µmol(f.u.) L^−1^. The viable cells that exclude the stain and the dyed dead cells were manually counted. Upon the application of the 5.3 µmol(f.u.) L^−^^1^ dose, the proliferation ability and the number of living MCF-7 cells were likely decreased in the cells treated with MZF@sil@Au-MTAB nanoshells and were significantly (*p* ≤ 0.05) decreased in the cells treated with MZF@sil@Au-MUA (Figure 7a). Despite such rather weak antiproliferative effects observed for the highest concentration of the nanoshells functionalized with MUA and MTAB, the proliferation of other treated cells did not differ significantly from the negative control. Importantly, the percentage of viable cells enumerated as alive in the trypan blue assay remained unaffected for all the treatments (Figure 7b). 

**Figure 8:**
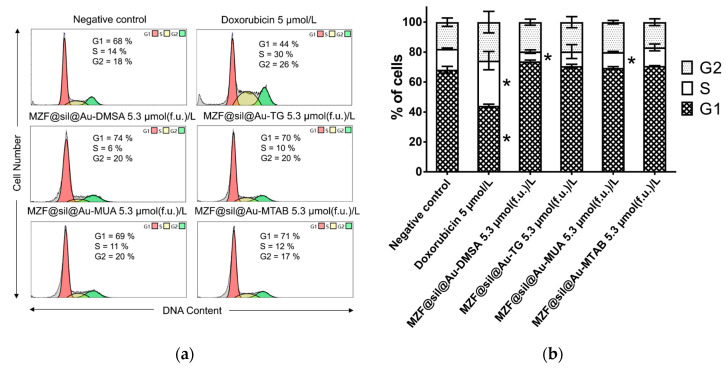
Analysis of the cell cycle of MCF-7 cells 48 h after the application of suspensions of gold nanoshells functionalized with DMSA, TG, MUA, and MTAB with a ferrite concentration of 5.3 µmol(f.u.) L^−1^: (**a**) Representative histograms with the mean percentage of cells cycling through phases G1, S, and G2 from flow cytometry of three separate treatments; (**b**) the bar graph summarizing the percentage of cells in each phase of the cell cycle. Data are presented as mean values ± SD from three experiments; the asterisk marks the results significantly different (*p* ≤ 0.05) from the negative control that was treated with sterile deionized water. Cells treated with 5 µM doxorubicin were used as a positive control.

Impaired proliferation frequently occurs as a consequence of cell cycle perturbations. Therefore, the cell cycle distribution of MCF-7 cells was examined upon exposure to gold nanoshells at the highest concentration used in the previous experiments, i.e., 5.3 µmol(f.u.) L^−1^ (Figure 8). The incubation of the cells with the nanoshells functionalized with DMSA and MUA for 48 h resulted in a slightly lower percentage of cells in the S-phase, 6% and 11%, respectively, compared with the untreated control having 14% of S-phase cells. In contrast, regarding the cells treated with doxorubicin as the positive control, the percentage of cells in the S-phase increased and in the G1-phase decreased. The cell cycle distribution in cells incubated with nanoshells functionalized with TG and MTAB did not differ significantly from the negative control.

### 3.3. Gold Nanoshells for Multimodal Imaging and pH Sensing

**Figure 9:**
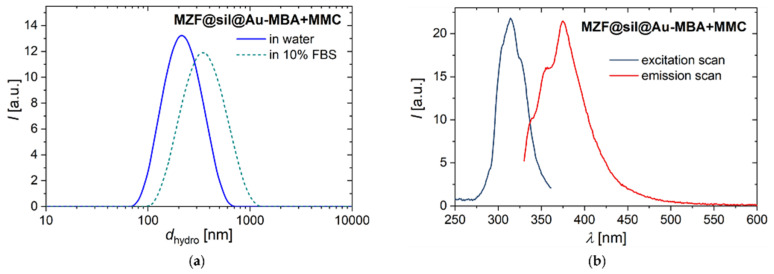
(**a**) The intensity-weighted hydrodynamic size distribution of MZF@sil@Au-MBA+MMC nanoshells in pure water and 10 vol% FBS measured by DLS. (**b**) Fluorescence spectra of MZF@sil@Au-MBA+MMC particles in an aqueous suspension with pH = 7. The excitation scan was recorded at λ_em_ = 375 nm, and the emission scan was measured with λ_ex_ = 314 nm.

The nanosized probe suggested for multimodal imaging and sensing of pH, i.e., the MZF@sil@Au-MBA+MMC nanoshells were subjected to DLS measurements in pure water and 10 vol% FBS to comparatively probe possible effects of conditions within a biological system, at least based on such a simplified model. Importantly, the measurements in the FBS suspension were repeatable (according to six consecutive measurements after the sample preparation) and did not indicate colloidal instability or time-dependent effects. Representative intensity-weighted distributions of the hydrodynamic size are shown in Figure 9a for both the suspensions. However, the hydrodynamic size increased from d_hydro,Z_ = 193 nm (pdi = 0.182) in pure water to d_hydro,Z_ = 272 nm (pdi = 0.290) in 10 vol% FBS, which can be primarily attributed to the adsorption of serum proteins to the surface of nanoshells, i.e. the formation of a protein corona. 

Fluorescence spectra of gold nanoshells functionalized with the combination of MBA and MMC are depicted in Figure 9b, showing maxima of λ_max,ex_ = 314 nm and λ_max,em_ = 375 nm in the excitation and emission scans, respectively. In spite of the relatively low fraction of the fluorophore employed in the functionalization mixture compared to the pH-sensitive SERS reporter (molar ratio of MMC: MBA of 1:5), these measurements demonstrate the clear fluorescence of MZF@sil@Au-MBA+MMC particles. Moreover, their spectra are consistent with data reported for MMC in the literature, e.g., λ_max,em_ = 374 nm was determined on MMC solutions both in dichloromethane and toluene [30].

^1^H NMR relaxometry showed that the MZF@sil@Au-MBA+MMC nanoshells represent an extremely efficient T_2_ contrast agent for MRI. At the temperature of 23 °C and in the magnetic field of 0.47 T, the longitudinal relaxivity was low, r_1_ = 3.309(3) s^−1^ mmol^−1^(f.u.) L, but a very high transverse relaxivity r_2_ of 903(28) s^−1^ mmol^−1^(f.u.) L was observed. 

The low value of r_1_ was expected since the thick silica and gold shells hinder water molecules from direct contact with the magnetic cores, and thus effectively eliminate the inner-sphere relaxation mechanism. At the same time, the distance from the magnetic core lowers the outer-sphere term. 

The high value of r_2_ relaxivity suggests that the nanoparticles influence ^1^H relaxation under the terms of the static dephasing regime (SDR) [31]. In this regime, which is valid for large particles, the ^1^H spins of water molecules in the suspension, diffusing during the NMR pulse sequence, experience only minor variations of local magnetic fields in the vicinity of the large particles. Therefore, the refocusing of the ^1^H spins during the CPMG sequence is not efficient and r_2_ reaches its higher limit. Importantly, SDR can be reached not only in the case of large crystallite sizes of the magnetic phase [32] but also clusters of smaller magnetic particles coated with a rather thick diamagnetic shell, as was experimentally demonstrated, e.g., for clusters of ≈11 nm-sized Mn-Zn ferrite nanoparticles encapsulated in TiO_2_, achieving the relaxivity of r_2_ ≈ 927 s^−1^ mmol^−1^(f.u.) L in the field of 0.47 T at room temperature [28].

**Figure 10:**
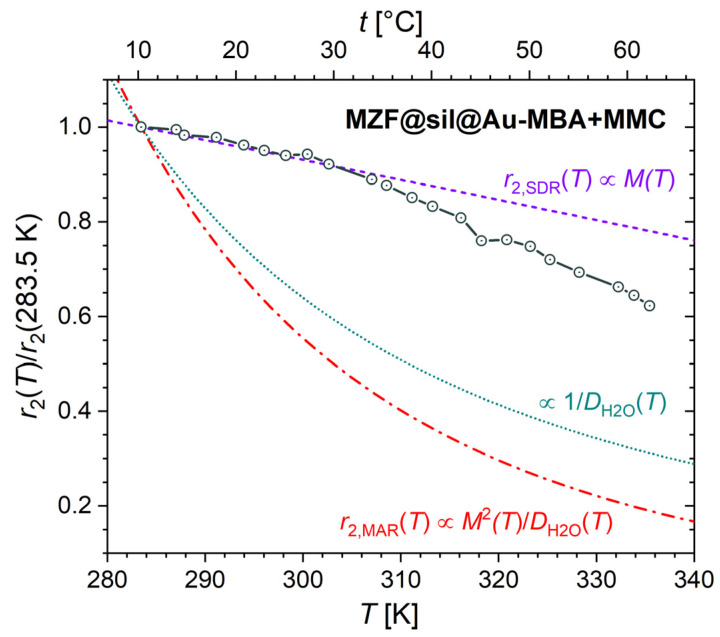
Temperature dependence of transverse relaxivity, r_2_, normalized by using the value at the lowest experimental temperature, r_2_(283.5 K), for MZF@sil@Au-MBA+MMC nanoshells in the magnetic field of 0.47 T. The dependence is compared with the dependences predicted for SDR (r_2,SDR_) and MAR (r_2,MAR_) and with the temperature dependence of the inverse value of the self-diffusion coefficient of water 1/D_H_2_O_(T).

To unambiguously identify the dominant relaxation regime, the transverse relaxivity was measured depending on temperature and compared with the dependences predicted for the SDR and motional averaging regime (MAR), which both can occur under the given experimental conditions but whose temperature dependences differ. The transverse relaxivity in SDR should follow, under certain assumptions, the temperature dependence of magnetization: r_2,SDR_(T) ∝ M(T). In contrast, the relaxivity in MAR is described by r_2,MAR_(T) ∝ M(T)^2^/D_(H_2_O)_(T) and is usually shaped mainly by the 1/D_H_2_O_(T) dependence, which is more pronounced than M(T) [28]. Thus, the experimental r_2_(T) dependence was normalized by using its value at the lowest experimental temperature of 10.3 °C and was compared with analogically normalized temperature dependences of M(T), 1/D_H_2_O_(T), and M(T)^2^/D_H_2_O_(T), where M(T) is the temperature dependence of magnetization of bare MZF cores measured at 0.47 T (data obtained from [28]) and D_H_2_O_(T) is the temperature dependence of the self-diffusion coefficient of water (calculated using the Speedy-Angel power law with parameters according to [33]). The results in Figure 10 confirm that the transverse relaxation in the studied suspension corresponds to SDR while the deviation from SDR at higher temperatures may be attributed to an increasing fraction of particles in MAR due to the growing self-diffusion coefficient and decreasing characteristic diffusion correlation time.

**Figure 11:**
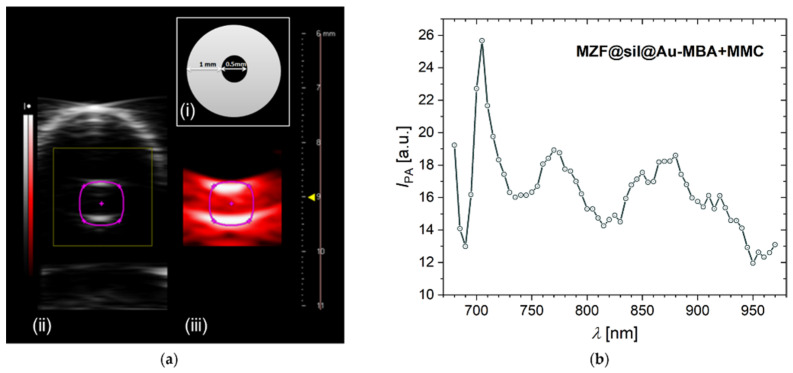
Photoacoustic study on the suspension of MZF@sil@Au-MBA+MMC nanoshells with a ferrite concentration of 0.64 mmol(f.u.) L^−1^. (**a**) PAI study: (i) A scheme showing the experimental setup—a cross-section of the tube filled with the suspension, (ii) an ultrasound image of the tube with the nanoshells, and (iii) a photoacoustic image of the same tube obtained at 680-nm excitation; (**b**) photoacoustic spectrum of the MZF@sil@Au-MBA+MMC nanoshells.

The MZF@sil@Au-MBA+MMC nanoparticles were further subjected to a proof-of-concept PAI study. In addition to the imaging of thin tubes filled with the suspension with a concentration of 0.64 mmol(f.u.) L^−1^ (~5.5 mg L^−1^ of gold based on magnetometry) by both ultrasound and PAI modalities of the employed bimodal platform (Figure 11a), photoacoustic spectrum was recorded in the range of 680–970 nm (Figure 11b). The spectrum proved a high signal yield in the NIR range, and the photoacoustic signal exhibited a maximum at around 700 nm, where the plasmonic resonance absorption occurs, as shown by the UV-Vis spectrum of MZF@sil@Au-MBA+MMC in Figure 4. However, the maximum in the photoacoustic spectrum is narrower compared to the broad band in the UV-Vis spectrum. This difference can be rationalized considering that the UV-Vis spectrum also includes the component of optical scattering, the maximum of which is red-shifted with respect to the absorption, whereas the photoacoustic spectrum primarily reflects the absorption (see, e.g., data on gold nanoshells with a diameter of ~60–70 nm and thickness of the shell 8–12 nm in the study [34]). 

In the context of extensive data on gold nanostructures reported in the literature, gold nanoshells seem to be especially convenient for PAI since their SPR wavelength can be tuned by adjusting their diameter and shell thickness to fit the NIR window, in which the light attenuation by living tissues is relatively low. For an ideal smooth gold nanoshell, a red shift of the SPR is predicted when increasing the ratio of its inner and outer diameters [35]. A red shift also occurs when increasing the shell thickness at a constant ratio of the two diameters [36]. In contrast, the SPR of gold nanospheres occurs at smaller wavelengths, typically below 600 nm (for example, λ_SPR_ = 575 nm for nanospheres with a diameter of ≈100 nm [37]) and increases only moderately with the particle size [36]. Gold nanorods offer similar versatility when it comes to adjusting λ_SPR_ to the NIR window when compared with gold nanoshells. The longitudinal SPR wavelength follows a linear dependence on the aspect ratio of the nanorods [36,38], and λ_SPR_ = 700 nm roughly corresponds to the aspect ratio of 3 in the work by Ni et al. [38]. Nevertheless, both nanorods and nanospheres lack the magnetic component of the present gold nanoshells with Mn-Zn ferrite cores.

**Figure 12:**
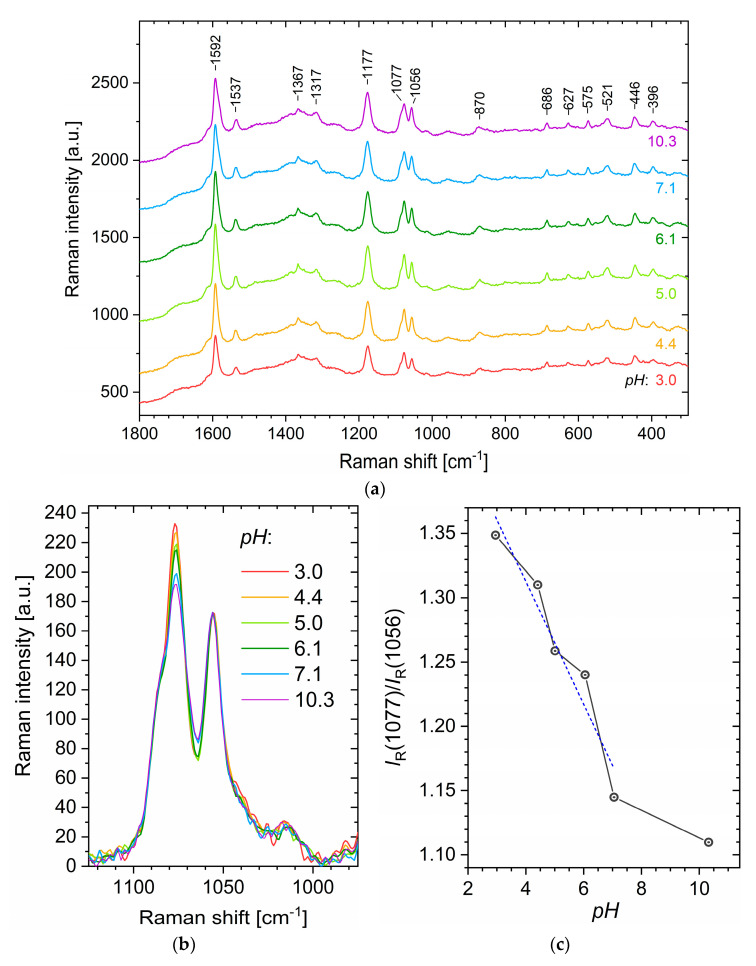
SERS study on aqueous suspensions of MZF@sil@Au-MBA+MMC nanoparticles whose pH was adjusted in the range of 3–10 using diluted NaOH or HCl. (**a**) Raw (unprocessed) SERS spectra measured on aqueous suspensions with varying pH and (**b**) detail of the 1125–975 cm^−1^ region normalized to the MMC band at 1056 cm^−1^ with the pH-dependent intensity of the MBA band at 1077cm^−1^. (**c**) The dependence of the ratio of Raman band intensities at 1077 cm^−1^ and 1056 cm^−1^ on pH, complemented by linear fit in the range of pH = 3–7.

The SERS spectra measured on aqueous suspensions of MZF@sil@Au-MBA+MMC nanoshells with different pH values are shown in Figure 12a, and the following interpretation of Raman bands is based on several reports [22,39,40,41,42]. The most intense band at ≈1592 cm^−1^ corresponds primarily to the aromatic ring vibration of MBA [39], which was present in fivefold molar excess to MMC, but involves also the in-plane C=C stretching of the lactone/benzene rings in MMC. The successful functionalization of gold nanoshells with MMC molecules is unambiguously evidenced by the band at ≈1537 cm^−1^, where modes of the benzene ring of MMC are manifested. The second most intense peak at ≈1177 cm^−1^ probably results from modes of both MMC and MBA [22]. Similarly, the peak at ≈1077 cm^−1^ can be ascribed to the ring vibrations primarily of MBA and to a lesser extent also of MMC, whereas the peak at 1056 cm^−1^ can be attributed just to MMC (probably the bending vibration of C-H and deformation vibration of C-O) [22].

Previous studies have already shown that the intensities of certain bands in the SERS spectra of MBA are dependent on pH and can be employed for pH sensing [12]. In the present case, the spectra are more complex due to the manifestation of MMC modes, which, however, can be employed as an internal standard for the normalization of the spectra. Avoiding the spectral region where glass typically contributes to an increased background and trying to analyze intense bands with a high signal-to-noise ratio, we will arrive at the region of 1235–975 cm^−1^ containing three intense peaks. Among them, the bands at ≈1077 cm^−1^ and 1056 cm^−1^ seem to be particularly suitable as revealed upon the normalization of the spectra to the MMC band at 1056 cm^−1^ (Figure 12b). The ratio of their intensities I_R_(1077)/I_R_(1056) shows a clear dependence on pH and decreases with increasing pH (Figure 12c). It would be too speculative to explain the observed dependence without DFT studies, but one may draw attention to, for example, the study by Michota and Bukowska [39], who suggested that MBA molecules are at least tilted with respect to the Ag/Au surface at neutral and higher pH, while they adopt more vertical orientation under more acidic pH.

## 4. Conclusions

The present study demonstrates that gold nanoshells with silica-coated magnetic cores formed by clusters of Mn-Zn ferrite nanoparticles provide a promising platform for multimodal imaging combined with sensing for biomedical and biological applications. The employed magnetic cores (≈30 nm clusters of ≈12 nm Mn_0.6_Zn_0.4_Fe_2_O_4_ crystallites) and gold nanoshells (≈127 nm and thickness of ≈27 nm) exhibit excellent contrast properties for MRI and PAI, namely extraordinarily high transverse relaxivity (≈900 s^−1^ mmol(f.u)^−1^ L based on the ferrite formula units) and a strong photoacoustic signal in NIR. Suitable organic functionalization, e.g., by fluorescent molecules and pH-sensitive SERS reporters, extends their applicability in fluorescence techniques and the sensing of pH via SERS measurements. Importantly, the functionalization of the gold surface of nanoshells with four different model molecules that varied the charge and surface chemistry did not lead to relevant cytotoxic effects or significant perturbation of cell cycle according to in vitro studies (MCF-7 cells, concentrations up to 5.3 µmol(f.u) L^−1^, i.e., monitoring up to 72 h upon treatment).

## Data Availability

Data are available on request.
